# Interfacial Voids Trigger Carbon-Based, All-Inorganic CsPbIBr_2_ Perovskite Solar Cells with Photovoltage Exceeding 1.33 V

**DOI:** 10.1007/s40820-020-00425-1

**Published:** 2020-04-06

**Authors:** Weidong Zhu, Zeyang Zhang, Dandan Chen, Wenming Chai, Dazheng Chen, Jincheng Zhang, Chunfu Zhang, Yue Hao

**Affiliations:** 1 State Key Discipline Laboratory of Wide Band Gap Semiconductor Technology and Shaanxi Joint Key Laboratory of Graphene, School of Microelectronics, Xidian University, Xi’an, 710071 People’s Republic of China; 2 College of Science, Xi’an Shiyou University, Xi’an, 710065 Shaanxi People’s Republic of China

**Keywords:** All-inorganic perovskite solar cells, CsPbIBr_2_, Photovoltage, Interfacial engineering, Stability

## Abstract

A novel interface design is proposed for carbon-based, all-inorganic CsPbIBr_2_ perovskite solar cells (PSCs) by introducing interfacial voids between TiO_2_ electron transport layer and CsPbIBr_2_ absorber. Compared with the general interfacial engineering strategies, this design exempts any extra modification layer in final PSC. More importantly, the interfacial voids produced by thermal decomposition of 2-phenylethylammonium iodide trigger three beneficial effects. First, they promote the light scattering in CsPbIBr_2_ film and thereby boost absorption ability of the resulting CsPbIBr_2_ PSCs. Second, they suppress recombination of charge carriers and thus reduce dark saturation current density (*J*_0_) of the PSCs. Third, interfacial voids enlarge built-in potential (*V*_bi_) of the PSCs, awarding increased driving force for dissociating photo-generated charge carriers. Consequently, the PSC yields the optimized efficiency of 10.20% coupled with an open-circuit voltage (*V*_oc_) of 1.338 V. The *V*_oc_ achieved herein represents the best value among CsPbIBr_2_ PSCs reported earlier. Meanwhile, the non-encapsulated PSCs exhibit an excellent stability against light, thermal, and humidity stresses, since it remains ~ 97% or ~ 94% of its initial efficiency after being heated at 85 °C for 12 h or stored in ambient atmosphere with relative humidity of 30–40% for 60 days, respectively.

**Electronic supplementary material:**

The online version of this article (10.1007/s40820-020-00425-1) contains supplementary material, which is available to authorized users.

## Highlights

- A novel interface design of producing interfacial voids is proposed for CsPbIBr_2_ perovskite solar cells (PSCs), which is free of any extra modification layer.
- Interfacial voids improve absorption of CsPbIBr_2_ film, reduce saturation current density, and enlarge built-in potential of the PSCs.
- The PSC yields a superior efficiency of 10.20% with a record-high photovoltage of 1.338 V.

**Electronic supplementary material:**

The online version of this article (10.1007/s40820-020-00425-1) contains supplementary material, which is available to authorized users.

## Graphical abstract

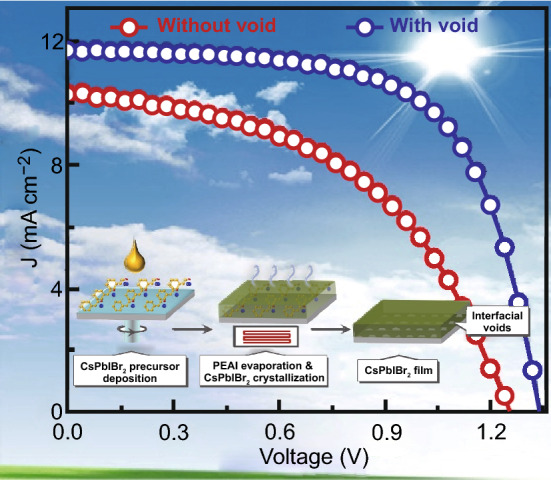

## Introduction

Organic–inorganic hybrid perovskite semiconductors have emerged as promising absorber materials for solar cells that are known as perovskite solar cells (PSCs), owing to their excellent optoelectronic properties, low cost, and versatile low-temperature processability of thin films [1–4]. Furthermore, the certified power conversion efficiency (PCE) of PSCs has reached up to 25.2% [5], approaching that of commercial crystalline silicon solar cells. However, the poor stability of organic–inorganic hybrid perovskite films under thermal, humidity, and light stresses brings a severe reliability issue to PSCs [3, 4, 6]. In addition, most of high-efficiency PSCs generally contain expensive and unstable organic hole transport layers (HTLs) coupled with noble metal electrodes. These functional films not only aggravate the instability problem but also burden the production cost of corresponding PSCs [3, 6, 7]. Hence, there is a growing attention to carbon-based, all-inorganic PSCs that avoid use of any HTLs and noble metal electrodes, in view of their multiply superiorities including upgraded stability, cost-effectiveness, and facile fabrication [7–11].

Currently, the absorber materials explored for carbon-based, all-inorganic PSCs largely include CsPbI_3_ [12, 13], CsPbI_2_Br [14–16], CsPbIBr_2_ [8, 17–20], and CsPbBr_3_ [7, 9, 21–23]. Among them, CsPbIBr_2_ holds the relatively well-balanced characters of bandgap and phase stability [8, 17–19, 24, 25]. Meanwhile, CsPbIBr_2_ is also applicable to build photovoltaic windows [26], semitransparent solar cells [27], water splitting systems [28], and tandem solar cells in conjunction with the state-of-the-art photovoltaic technologies [11]. Nevertheless, the PCEs of carbon-based, all-inorganic CsPbIBr_2_ PSCs are still far from their theoretical level [29, 30], and even lag behind that of the ones based on other analogs [13, 15, 23]. This dilemma can be largely attributed to their inferior open-circuit voltage (*V*_oc_) and fill factor (FF) values, which are eventually caused by the serious non-radiative recombination within CsPbIBr_2_ films and interfacial recombination in the PSCs.

To solve the above obstacles, great efforts have been dedicated in improving the quality of CsPbIBr_2_ films, targeting to suppress non-radiative recombination of charge carriers, by crystallization engineering or heteroatom replacement on Cs site or Pb site [19, 20, 25, 31]. For example, our previous work [19] demonstrated that modification of general one-step method with an intermolecular exchange reaction could produce a pinhole-free CsPbIBr_2_ film composed of micro-sized, [100] preferential grains, which finally enabled an optimized PCE of 9.16%. More recently, it was reported that Ba(II) doping could boost the grain sizes and crystallinity of CsPbIBr_2_ film, bringing out reduced trap states and suppressed non-radiative recombination. As a result, the PCE of corresponding PSC was boosted from 8.40 to 10.51% [31].

Interfacial modification with extra functional materials has been attempted to weaken interfacial recombination of charge carriers and therefore improve the PCEs of CsPbIBr_2_ PSCs [17, 18, 30, 32]. For example, the innovative modification of TiO_2_ electron transport layer (ETL) with CsBr or SmBr_3_ could strikingly improve the PSC’s PCE to over 10% coupled with desirable FF of ~ 0.70, which represents the highest PCE among CsPbIBr_2_ PSCs reported to date [18, 30]. Guo et al. [17] reported that surficial passivation of SnO_2_ ETL could enable the CsPbIBr_2_ PSCs with a record-high *V*_oc_ of 1.31 V in spite of its low PCE of 2.06%. Clearly, these progresses verify the considerable feasibility of interfacial modification strategy in overcoming the obstacle faced by carbon-based, all-inorganic CsPbIBr_2_ PSCs. However, the extra interfacial modifiers inevitably complex the configuration of CsPbIBr_2_ PSCs and thus bring some new parasitic issues. For example, the modifiers multiply the interfaces of CsPbIBr_2_ PSCs, leading to an increased degradation risk [3, 33, 34]. Meanwhile, they will also alter the crystallization dynamics of atop CsPbIBr_2_ films [35, 36], which brings a new challenge to prepare high-quality CsPbIBr_2_ film. Thus, it is highly desired to develop more feasible interfacial engineering strategy to boost interfacial kinetics of charge carriers in carbon-based, all-inorganic CsPbIBr_2_ PSCs, thereby boosting its *V*_oc_ and FF as well as PCE.

In this study, we propose an interfacial engineering strategy for carbon-based, all-inorganic CsPbIBr_2_ PSCs by making interfacial voids between TiO_2_ ETL and CsPbIBr_2_ film. This strategy avoids introducing any extra modification layer in ultimate PSCs compared with the conventional ones. Further, we reveal that the interfacial voids can stimulate enhanced absorption, decreased interfacial recombination of charge carriers, as well as enlarged built-in potential (*V*_bi_) in resulting CsPbIBr_2_ PSCs. Hence, the optimized one yields the outstanding PCE of 10.20% coupled with a high *V*_oc_ of 1.338 V, a FF of 0.65, and a short-circuit current density (*J*_sc_) of 11.73 mA cm^−2^. The PCE is beyond nearly most of CsPbIBr_2_-based cells reported earlier, and particularly the *V*_oc_ represents the highest level among them. Moreover, the PSC exhibits excellent photo-stability as well as long-term stability against thermal and humidity stresses.

## Experimental Section

### Materials and Reagents

Patterned FTO glass substrates (Pilkington, TEC-8, 8 Ω sq^−1^) were supplied by Yingkou OPV Tech New Energy Co., Ltd., China. Conductive carbon paste was bought from Shanghai MaterWin New Materials Co., Ltd., China. Lead bromide (PbBr_2_, ultra-dry, 99.999%), cesium iodide (CsI, ultra-dry, 99.998%), and dimethyl sulfoxide (DMSO, anhydrous, 99.8 +%), isopropanol (anhydrous, 99.5 +%) are received from Alfa-Assar. 2-phenylethylammonium iodide (PEAI, 99.99%) was purchased from Xi’an Polymer Light Technology Co., Ltd., China. All of them are used without further treatment or purification.

### Preparation of PEAI-Loaded FTO/TiO_2_ Substrate

Firstly, patterned FTO glass with size 2 × 2.5 cm^2^ was washed with detergent, ultrapure water, acetone, and ethanol in an ultrasonic cleaner successively. Next, TiO_2_ sol was spin coated onto the FTO glass at 3500 rpm for 30 s. After being annealed at 500 °C for 60 min in ambient air, the FTO/TiO_2_ substrate was achieved. Finally, 100 μL 3 mg mL^−1^ PEAI in isometric deionized water and isopropanol was spin-coated on the substrate at 3500 rpm for 60 s and baked at 80 °C for 5 min in ambient air in order to load PEAI species onto FTO/TiO_2_ substrate.

### Deposition of CsPbIBr_2_ Film

CsPbIBr_2_ precursor (1.0 M) was firstly prepared by dissolving 370.0 mg PbBr_2_ and 260.0 mg CsI in 1 mL DMSO at room temperature with vigorously stirring. Then, the PEAI-loaded FTO/TiO_2_ substrate was transferred into a N_2_-filled glovebox. And, 70 μL CsPbIBr_2_ precursor was spin-coated the substrate at 1500 rpm for 20 s and 5000 rpm for 60 s. After the spin-coating process, the sample was annealed at 280 °C for 15 min to form the crystallized CsPbIBr_2_ film. For comparison, a CsPbIBr_2_ film on FTO/TiO_2_ substrate without loading of PEAI species was also prepared according to the same procedures above.

### Fabrication of Carbon-Based, All-Inorganic PSC

Carbon paste with the area of 0.10 cm^2^ was firstly coated on CsPbIBr_2_ film by screen-printing method. Then, the sample was heated at 120 °C for 15 min to promote evaporation of solvent of carbon paste. After cooling down naturally to room temperature, the carbon-based, all-inorganic PSC was achieved for further characterizations.

## Results and Discussion

### Formation Mechanism of CsPbIBr_2_ Films with Interfacial Voids

**Scheme 1:**
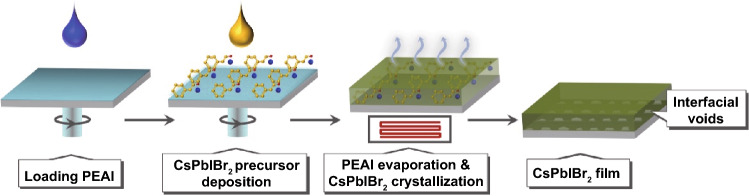
Schematic procedures for preparation of CsPbIBr_2_ film with interfacial voids

The main preparation procedures of CsPbIBr_2_ film with interfacial voids are shown in Scheme 1. To be brief, FTO/TiO_2_ substrate was firstly prepared according to the recipe described in our previous work [18, 19]. Then, 2-phenylethylammonium iodide (PEAI, C_6_H_5_CH_2_CH_2_NH_3_I) in isometric deionized water and isopropanol was deposited on FTO/TiO_2_ substrate by spin-coating method, with the aim of loading PEAI species on TiO_2_ surface. The successful loading of PEAI can be verified by the scanning electron microscope (SEM) results in Fig. S1. Next, CsPbIBr_2_ precursor was spin-coated onto the substrate by one-step spin-coating method. Finally, the sample was annealed at 280 °C for 15 min to promote the crystallization of CsPbIBr_2_ grains. It is noted that the release of PEAI species happened simultaneously during this high-temperature annealing stage, as discussed below.

**Fig. 1:**
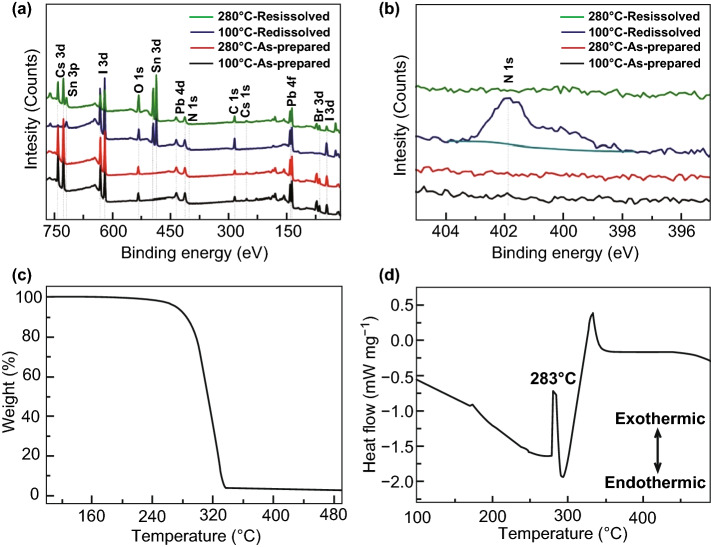
**a** XPS survey spectra of as-prepared and in situ redissolved CsPbIBr_2_ films annealed at 100 °C and 280 °C, **b** corresponding core-level N 1*s* XPS spectra, **c** TGA heating curve of PEAI powder expressed as weight% as a function of applied temperature, **d** DSC curve of PEAI powder

We conducted X-ray photoelectron spectroscopy (XPS) measurement to examine the constituents of obtained CsPbIBr_2_ film. For comparison, a same CsPbIBr_2_ film that was prepared at a low annealing temperature of 100 °C was also investigated. The reason for adopting this low annealing temperature to prepare the control sample is that it cannot cause the sublimation or decomposition of PEAI molecules, as demonstrated previously [37, 38]. Figure 1a manifests that clear XPS peaks corresponding to Cs, Pb, I, and Br elements appear for the films annealed at either 280 or 100 °C, indicating that they are composed of CsPbIBr_2_ materials. However, no any signal corresponding to PEAI species can be measured from them, even though the FTO/TiO_2_ substrate contains PEAI species. The core-level N 1*s* XPS spectra in Fig. 1b further verify this fact, since the typical N 1*s* peak of PEAI species is absent [38].

In general, XPS measurement can only reflect surficial composition information of samples. To insight bulk composition of the concerned CsPbIBr_2_ films, they were redissolved in situ by *N*,*N*-dimethylformamide (DMF) solvent and annealed again at 90 °C for 10 min to evaporate the solvent away. As shown in Fig. 1a, after such treatment the additional Sn 3*d* peaks that originate from FTO/TiO_2_ substrate can be detected from both films, indicating the full dissolution of CsPbIBr_2_ films by DMF solvent and therefore the possible non-uniform composition distribution in the films or their interfaces is eliminated. In this manner, XPS measurement can be used to study bulk composition of CsPbIBr_2_ films reasonably. Once again, as exhibited in Fig. 1b, the N 1*s* peak located at 401.8 eV can be detected from CsPbIBr_2_ film prepared at 100 °C, manifesting the existence of PEAI species in it [37, 38]. By contrast, the peak is still missing in CsPbIBr_2_ film prepared at 280 °C, indicating that there is no detectable PEAI species in it indeed. This can be also supported by the energy-dispersive spectrometer (EDS) result in Fig. S2. We attribute such phenomenon to the sublimation of PEAI species during the high-temperature annealing stage of CsPbIBr_2_ film.

To support our standpoint, thermal property of PEAI powder was investigated by thermogravimetric analysis (TGA) and differential scanning calorimetry (DSC) [39, 40]. The TGA curve in Fig. 1c indicates that PEAI powder undergoes 100% weight loss in one step with the onset temperature of ~ 185 °C, suggesting that it is sublimated without complex decomposition procedure. This can be supported by the sharp transition step in corresponding differential TGA curve in Fig. S3. Moreover, the endothermic peak at ~ 283 °C in the DSC curve in Fig. 1d also suggests that the release of PEAI is related to a sublimation process. Thus, the typical sublimation temperature of PEAI powder is estimated to be ~ 283 °C, which is nearly the same as the annealing temperature of CsPbIBr_2_ film adopted in this work. Additionally, it is noted that the DSC measurement was performed on PEAI powder. In the case of within CsPbIBr_2_ thin film, a much larger surface-to-volume ratio is provided; therefore, the sublimation temperature could be lowered considerably to below ~ 283 °C for PEAI. Thus far, all the above observations provide conclusive evidences that in terms of CsPbIBr_2_ film prepared at 280 °C the original PEAI species on FTO/TiO_2_ substrate do not reside in its bulk or at its interface, but are sublimated during the thermal annealing stage.

### Morphology Characterization

**Fig. 2:**
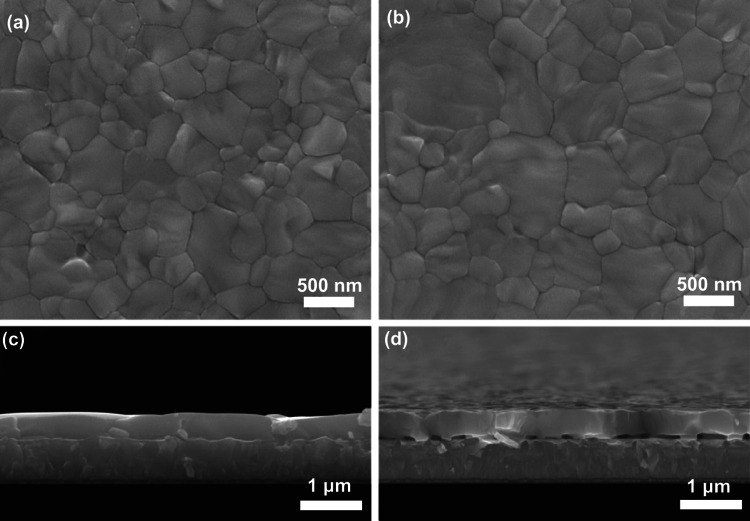
Top-view and cross-sectional SEM images of CsPbIBr_2_ films deposited on FTO/TiO_2_ substrates: **a**, **c** without and **b**, **d** with loading of PEAI species

With this fact in mind, we turned to study the microstructure of CsPbIBr_2_ films deposited on FTO/TiO_2_ substrates without and with loading of PEAI species. Their top-view SEM images are provided in Fig. 2a, b. Clearly, both films are composed of closely packed crystalline grains without any discernible pinholes. Their grains are featured by dominant triple-junctions grain boundaries with equal vertex angle of 120°, implying that they are in equilibrium state with low internal stress [41, 42]. By contrast, the CsPbIBr_2_ film deposited on FTO/TiO_2_ substrate loaded with PEAI species exhibits larger grain sizes. The statistical results in Fig. S4 indicate its average grain size of ~ 576 nm, which is slightly larger than the one deposited on FTO/TiO_2_ substrate without loading of PEAI species (~ 475 nm). It should be noted that the formation of coarsened grains is mainly attributed to the decreased nucleation density of CsPbIBr_2_ grains, as a result of improved non-wetting of CsPbIBr_2_ precursor to FTO/TiO_2_ substrate induced by PEAI species (Fig. S5) [36, 43], along with the templating effect of PEAI species during the crystallization of CsPbIBr_2_ grains.

Figures 2c, d and S6 provide cross-sectional SEM images of the concerned CsPbIBr_2_ films. They reveal that the films have a similar thickness of ~ 400 nm. And, each of individual grains in the films is able to vertically penetrate entire film thickness. Such feature maybe endow excellent transport behavior of charge carriers along them, since the transport and transfer of charge carriers do so without traversing any grain boundaries in this case [4, 41]. By comparison, interfacial microstructure of these two films differs obviously. For the CsPbIBr_2_ film deposited on FTO/TiO_2_ substrate without loading PEAI species, the interface between CsPbIBr_2_ film and FTO/TiO_2_ substrate is contacted seamlessly. However, there are many voids that have the typical size of ~ 100 nm in the same interface, in terms of CsPbIBr_2_ film deposited on FTO/TiO_2_ substrate loaded with PEAI species. These voids give rise to the rough contact interface between CsPbIBr_2_ film and FTO/TiO_2_ substrate. It is also noted that because PEAI species are sublimated during the thermal annealing stage of CsPbIBr_2_ film, we conclude that the pre-deposited PEAI species likely act as pore-forming agent in the present case. The strategy of using pore-forming agent has been widely used to create pores in traditional ceramic materials [44, 45]. Our viewpoint can be further supported by the cross section SEM image of the same CsPbIBr_2_ film prepared with large concentration of PEAI solution (10 mg mL^−1^), as provided in Fig. S7. It is clear that the voids with much larger sizes are produced when the more PEAI species were pre-deposited. Overall, the PEAI species on FTO/TiO_2_ substrate, serving as pore-forming agent, can promote the formation of large-sized grains in ultimate CsPbIBr_2_ film as well as some voids at the interface between CsPbIBr_2_ film and FTO/TiO_2_ substrate.

### Crystalline and Optical Property Analysis

**Fig. 3:**
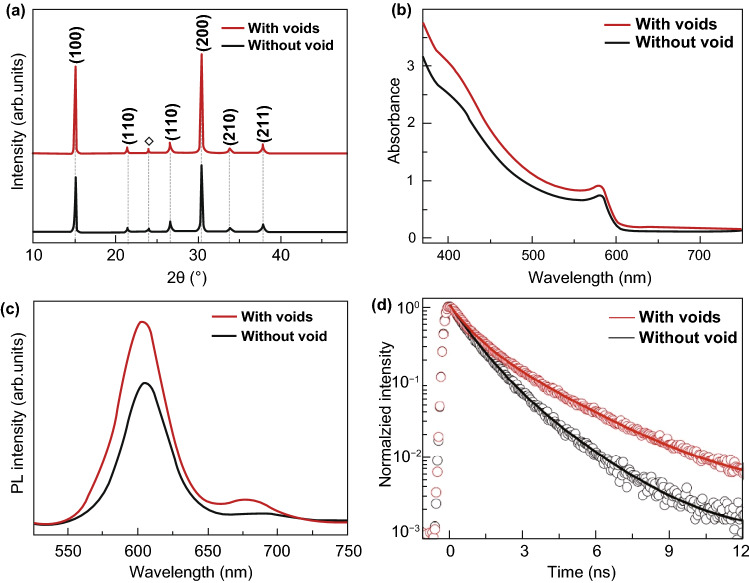
**a** XRD patterns and **b** UV–vis absorption spectra of CsPbIBr_2_ films without and with interfacial voids, **c** steady-state PL spectra and **d** TRPL curves of CsPbIBr_2_ films without and with interfacial voids on insulating glass substrates instead of FTO/TiO_2_ substrates

Next, the crystalline structure and optical properties of as-prepared CsPbIBr_2_ films are investigated. For clarification, the CsPbIBr_2_ film as well the cell deposited on FTO/TiO_2_ substrate without loading of PEAI species are labeled as “without void,” and the ones on FTO/TiO_2_ substrate with loading of PEAI species are named as “with voids” hereinafter. Figure 3a presents X-ray diffraction (XRD) patterns of CsPbIBr_2_ films without and with interfacial voids. Both samples exhibit the pure cubic perovskite phase, since the diffraction peaks at 15.1°, 21.4°, 26.6°, 30.4° 33.8°, and 37.8° are well consistent with those of (100), (110), (111), (200), (210), and (211) planes of CsPbIBr_2_ crystals reported previously [10, 26]. Meanwhile, the XRD peaks of (100) and (200) planes are dominantly intense for both samples, indicating that the grains in them have (100)-preferential orientation. For cubic perovskite polycrystalline films, {100} grains facets are generally along the direction perpendicular to FTO/TiO_2_ substrate, which well coincides the vertically penetrated grains morphology observed in the cros section SEM images in Figs. 2c, d and S6 for the concerned CsPbIBr_2_ films. In addition, the dominant diffraction peaks of CsPbIBr_2_ film with interfacial voids are more intense than the one without voids, and the full-width-at-half-maximum (FWHM) values of its (100) and (200) XRD peaks are smaller (Table S1). These results reveal the better crystallinity with fewer intragranular defects and less scattering of grain boundaries for the CsPbIBr_2_ film with interfacial voids, in consideration of the similar thickness of the two films [22, 41, 43]. Such features are in accordance with the larger grains of CsPbIBr_2_ film with interfacial voids.

Figure 3b presents the ultraviolet–visible (UV–vis) spectra of as-prepared CsPbIBr_2_ films. Both of them exhibit the typical absorption features of CsPbIBr_2_ materials including the exciton absorption peak at ~ 560 nm and absorption edge of ~ 599 nm that is in accordance with the bandgap of CsPbIBr_2_ (2.07 eV) [20, 25, 27]. In comparison, the CsPbIBr_2_ film with interfacial voids shows higher absorption intensities in the whole absorption ranges, revealing its better light absorption capacity. In principle, this desired feature mainly originates from either improved quality of CsPbIBr_2_ film or scattering effect of interfacial voids in it, in view of the similar thickness of the two films. To exclude the effect of the former, UV–vis characters of the concerned CsPbIBr_2_ films were collected from the side of FTO glass, wherein the detection beam streams from the top surface of CsPbIBr_2_ films. The results in Fig. S8 reveal that the differences of their absorption intensities decrease accordingly. That is to say, the better absorption ability of CsPbIBr_2_ film with interfacial voids observed in Fig. 3b largely comes from the beneficial scattering effect of interfacial voids, which may make great contribution to *J*_sc_ of ultimate PSC.

Figure 3c, d gives steady-state photoluminescence (PL) spectra and time-resolved PL (TRPL) curves of CsPbIBr_2_ films without and with interfacial voids grown on insulating glass substrates instead of FTO/TiO_2_ substrates, respectively. As shown in Fig. 3c, CsPbIBr_2_ films exhibit the dominant PL peaks at ~ 600 nm, in well agreement with their bandgaps. The shoulder peaks can be also observed for both samples, as indicated by the logarithmic-scale PL plots in Fig. S9. This phenomenon is mainly caused by light-induced halide phase segregation in CsPbIBr_2_ films, wherein some I-rich domains with low bandgap and Br-rich domains with wide bandgap are formed under illumination, as demonstrated in previous works [19, 46–48]. In addition, the CsPbIBr_2_ film with interfacial voids yields the stronger PL peak intensity, revealing the weaker non-radiative recombination of charge carriers in it. Figure 3d shows the corresponding TRPL results, which can provide semi-quantitative information about non-radiative recombination of charge carriers [2, 4, 9, 48]. The curves are fitted by a bi-exponential decay function of time (*t*) [2, 4]: $F(t) = \sum {A_{i} e^{{\tau /\tau^{i} }} ;\,i = 1,2}$, in which *A*_i_ and *t*_i_ are weight factor and time constant, respectively. In this manner, the average carrier lifetime (*t*_ave_) can be estimated according to the equation of $\tau_{\text{ave}} = \sum {A_{i} \tau_{i}^{2} } /\sum {A_{i} \tau_{i} } ;\,i = 1,2$. The CsPbIBr_2_ film with interfacial voids exhibits the *τ*_ave_ of 1.91 ns, which is higher than the one without interfacial voids (1.26 ns). The longer *τ*_ave_ of the former indicates the fewer defects in it, which is originally attributed to the larger grains and better crystallinity of CsPbIBr_2_ film with interfacial voids.

### Photovoltaic Performance

**Fig. 4:**
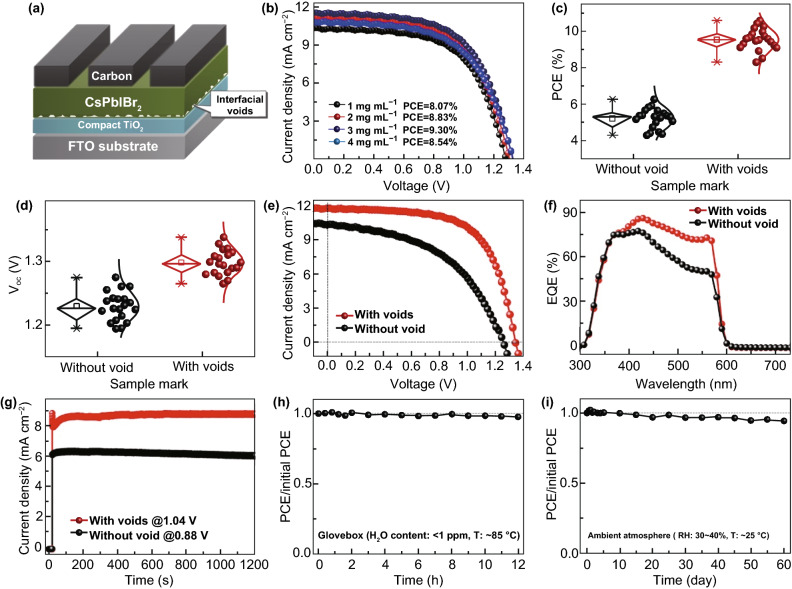
**a** Schematic layer stacking of carbon-based, all-inorganic CsPbIBr_2_ PSC, **b** typical *J*–*V* curves of the PSCs employed CsPbIBr_2_ films prepared with different concentrations of PEAI solutions, **c** statistic PCEs and **d** *V*_oc_ distributions of 20 independent PSCs based on CsPbIBr_2_ films without and with interfacial voids, **e** *J*–*V* curves, **f** EQE spectra, and **g** steady-state photocurrent outputs of the champion PSCs based on CsPbIBr_2_ films without and with interfacial voids. Time-dependent variations of PCEs of the un-encapsulated PSC based on CsPbIBr_2_ film with interfacial voids in **h** glovebox atmosphere and **i** ambient condition

Hitherto, we reveal that the CsPbIBr_2_ film with interfacial voids has slightly larger grains and higher crystallinity, which result in the weaker non-radiative recombination of charge carriers in it. Such feature is expected to improve the *V*_oc_ and FF of ultimate PSC [18, 30, 32, 49]. Meanwhile, the interfacial voids trigger intense light scattering effect [50], which gives rise to the much higher absorption of the CsPbIBr_2_ film and hence maybe boost the *J*_sc_ of corresponding PSC. It is generally recognized that interfacial voids are harmful to the performance of planar PSC, since they inevitably reduce the contact area between absorber film and charge transporting layer, thus increasing the transporting resistance of charge carriers. Even so, in the present case, we speculate that such negative effect can be fully counteracted by the multiply beneficial contributions of the interfacial voids described above. To verify our inference, the CsPbIBr_2_ films without and with interfacial voids are used to construct carbon-based, all-inorganic PSCs with the structure of FTO/TiO_2_/CsPbIBr_2_/Carbon as illustrated in Fig. 4a. Firstly, the PEAI solution concentration was optimized according to the performance of resulting PSCs. As shown in Fig. 4b, the PSC yields the optimized PCE when 3 mg mL^−1^ PEAI solution was used. So, this solution is adopted to fabricate all the PSCs hereinafter. Figure 4c provides the statistical PCEs of 20 independent PSCs based on the CsPbIBr_2_ films without and with interfacial voids, which were measured under simulated AM 1.5 G illumination (100 mW cm^−2^) and a scan rate of 10 mV s^−1^. A black mask containing a circular aperture with specific area of 0.085 cm^2^ was used to confine the active area of the PSCs. Accordingly, the average PCEs are estimated to be 5.22 ± 0.54% and 9.54 ± 0.59% for the PSCs fabricated with CsPbIBr_2_ films without and with interfacial voids, respectively. It is extremely surprised that the CsPbIBr_2_ films with interfacial voids can enable over 85% enhancement in average PCE of carbon-based, all-inorganic PSCs. Figure 4d shows the statistical *V*_oc_ values of as-fabricated PSCs, from which the average *V*_oc_ values are estimated to be 1.230 ± 0.023 and 1.297 ± 0.018 V for the PSCs based on CsPbIBr_2_ films without and with interfacial voids, respectively. Thus, we can conclude that the *V*_oc_ values of the PSCs based on CsPbIBr_2_ films with interfacial voids are larger consistently. It is worth noting that the superior *V*_oc_ is not only vital to overcome the PCE obstacle facing current CsPbIBr_2_ PSCs, but also can promote their applications in other energy conversion systems, such as water splitting cells [11, 17, 28].

Figure 4e displays the current density versus voltages (*J*–*V*) curves of the best-performing PSCs employed CsPbIBr_2_ films without and with interfacial voids, respectively. The typical photovoltaic parameters including *V*_oc_, FF, *J*_sc_, and PCE are provided in Table 1. The PSC fabricated with CsPbIBr_2_ film with interfacial voids delivers the *V*_oc_ of 1.338 V, FF of 0.65, and *J*_sc_ of 11.73 mA cm^−2^, leading to a much higher PCE of 10.20%. Such PCE is beyond nearly all the CsPbIBr_2_ PSCs reported previously. More importantly, as shown in Table 2, the *V*_oc_ achieved herein stands the highest value among them [8, 17–20, 25–27, 30, 31, 35, 47, 48, 51–56].

**Table 1:** Photovoltaic parameters of best-performing PSCs based on CsPbIBr_2_ films without and with interfacial voids

Cells	*V*_oc_ (V)	FF	*J*_sc_ (mA cm^−2^)	PCE (%)
Without void	1.254	0.49	10.31	6.34
With voids	1.338	0.65	11.73	10.20

**Table 2:** Summary of *V*_oc_ values and PCEs for CsPbIBr_2_ PSCs reported so far

Cell configuration	*E*_g_ (eV)	*V*_oc_ (V)	PCE (%)	Refs.
FTO/c-TiO_2_/CsPbIBr_2_/Carbon	2.07	1.338	10.20	This work
FTO/TiO_2_/SmBr_3_/Sm-doped CsPbIBr_2_/spiro-OMeTAD/Au	2.11	1.17	10.88	[30]
FTO/c-TiO_2_/CsBr/CsPbIBr_2_/Carbon	2.05	1.261	10.71	[18]
FTO/TiO_2_/CsPb(Ba)IBr_2_/spiro-OMeTAD/Au	~ 2.11	1.19	10.51	[31]
ITO/SnO_2_/S-doped CsPbIBr_2_/P3HT/Au	2.05	1.30	9.78	[25]
FTO/c-TiO_2_/CsPbIBr_2_/Carbon	2.05	1.245	9.16	[19]
FTO/c-TiO_2_/CsPbIBr_2_/Carbon	2.05	1.283	8.60	[51]
ITO/SnO_2_/CsPbIBr_2_/spiro-OMeTAD/Ag	2.00	1.15	8.54	[52]
FTO/c-TiO_2_/m-TiO_2_/CsPbIBr_2_/Carbon	1.90	1.08	8.25	[8]
FTO/c-TiO_2_/CsPbIBr_2_/spiro-OMeTAD/Au	2.05	1.227	8.02	[47]
ITO/SnO_2/_C60/CsPbIBr_2_/spiro-OMeTAD/Au	2.04	1.18	7.34	[48]
FTO/TiO_2_/CsPbIBr_2_/spiro-OMeTAD/Ag	2.07	1.28	7.31	[53]
ITO/Passivated SnO_2_/CsPbIBr_2_/Carbon	2.07	1.23	7.00	[17]
FTO/c-TiO_2_/CsPbIBr_2_/Carbon	2.05	1.142	6.55	[19]
FTO/c-TiO_2_/m-TiO_2_/CsPbIBr_2_/spiro-OMeTAD/Au	2.05	1.121	6.30	[54]
FTO/c-TiO_2_/m-TiO_2_/CsPbIBr_2_/Carbon	1.89	0.96	6.14	[20]
FTO/In_2_S_3_/CsPbIBr_2_/Spiro-OMeTAD/Ag	2.08	1.09	5.59	[27]
FTO/NiO_x_/CsPbIBr_2_/ZnO/Al	2.10	1.01	5.57	[26]
FTO/NiO_x_/CsPbIBr_2_/MoO_x_/Au	2.08	0.85	5.52	[35]
ITO/ZnO/CsPbIBr_2_/spiro-OMeTAD/Ag	2.06	1.04	4.8	[55]
FTO/c-TiO_2_/CsPbIBr_2_/Au	2.05	0.959	4.70	[56]

External quantum efficiency (EQE) spectra of the champion PSCs are given in Fig. 4f. Photocurrent response of each PSC is consistent with the absorption feature of CsPbIBr_2_ film. By contrast, the PSC based on CsPbIBr_2_ film with interfacial voids exhibits much higher EQEs in the wavelength regions from 400 to 580 nm, disclosing its better photoelectric conversion capability as a result of higher light absorption and more energetic extraction of charge carriers. Moreover, a remarkable EQE peak of 86% can be realized in the PSC. In addition, the integrated current densities from the EQE spectra (Fig. S10) are basically consistent with the *J*_sc_ provided in Table 1, wherein the discrepancies may be resulted from the absence of light soaking during EQE tests and the spectral mismatch [30, 31, 52].

We also tested the steady-state photocurrent outputs of the champion PSCs under maximum power-point conditions (Fig. 4g). They exhibit fast light-response, indicating smooth charge extraction. This desired character is mainly relied to large energy difference of conduction band minimums (CBMs) for CsPbIBr_2_ absorber and TiO_2_ ETL [18, 51]. One can see that the PSC based on CsPbIBr_2_ film without interfacial voids yields the stabilized current density of ~ 6.18 mA cm^−2^ under a constant bias voltage of 0.880 V, which corresponds to a stabilized PCE of 5.44%. And, the one without interfacial voids exhibits the stabilized PCE of 8.99% under 1.040 V, which supports the better performance of the PSC fabricated with CsPbIBr_2_ film with interfacial voids. However, both steady-state PCEs are smaller than those of extracted from *J*–*V* curves, indicative of inevitable anomalous *J*–*V* hysteresis. The *J*–*V* hysteresis is frequently observed in previous CsPbIBr_2_ PSCs and primarily related to light-induced phase segregation in CsPbIBr_2_ films [8, 46–48, 51, 52, 54, 55]. In addition, one can notice that during the test period the photocurrent outputs of both PSCs are stable in ambient atmosphere, revealing their excellent ability against humidity and light attacking.

Finally, thermal stability and long-term stability of the champion PSC prepared with CsPbIBr_2_ film with interfacial voids were studied. For thermal stability test, the un-encapsulated PSC was continuously heated at 85 °C on a hotplate in N_2_-filled glovebox. And, for long-term stability test the same PSC was stored at room temperature in ambient atmosphere with relative humidity of 30–40%. As shown in Fig. 4h, the PSC can remain ~ 97% of its initial PCE after being tested for 12 h, indicative of its superior thermal stability. We attribute this favored aspect to the excellent resistance of CsPbIBr_2_ film to high temperature, as it is prepared at a higher annealing temperature of 280 °C. In general, the annealing temperatures for its analogs including CsPbI_3_, CsPbI_2_Br, and CsPbBr_3_ are 200, 160, and 250 °C, respectively. Figure 4i provides the result of long-term stability test. The same PSC can sustain ~ 94% of its initial PCE after storing for 60 days, making clear its superior long-term stability against humidity and interface degradation. Overall, one can conclude that the CsPbIBr_2_ film with interfacial voids can enable the carbon-based, all-inorganic PSC with outstanding PCE and record-high *V*_oc_, along with excellent operation stability under light, thermal, or humidity stress.

### Carrier Dynamics

**Fig. 5:**
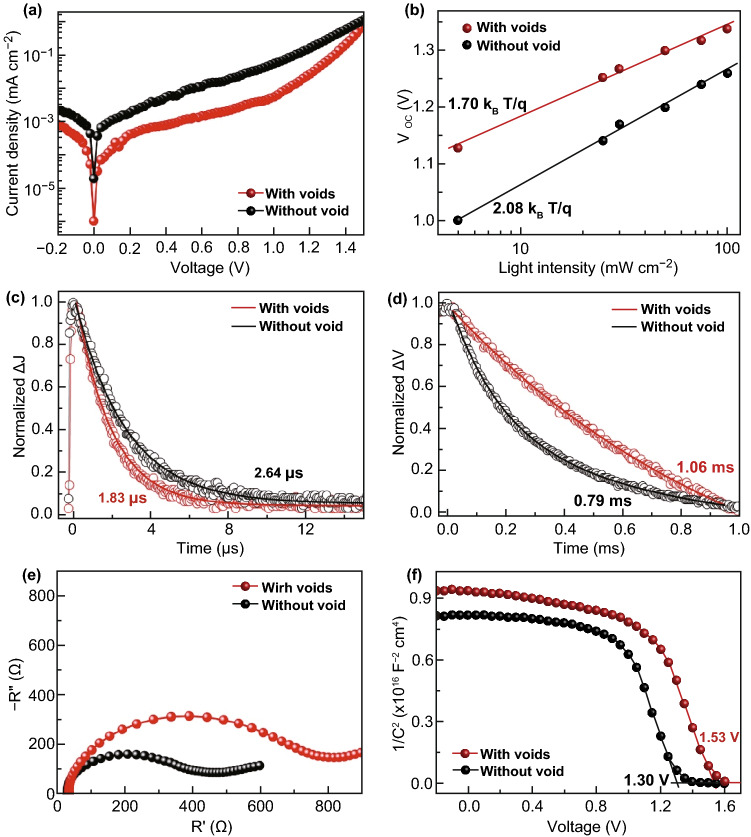
**a** Dark *J*–*V* curves, **b** the dependence of *V*_oc_ on light intensity, **c** TPC curves, and **d** TPV curves for the PSCs based on CsPbIBr_2_ films without and with interfacial voids, **e** Nyquist plots and **f** M–S plots of the same cells

To gain insight into the relationship between the carrier dynamics and performance of PSCs prepared with CsPbIBr_2_ films without and with interfacial voids, we explored the transport mechanisms in the fabricated PSCs by means of various characterization techniques, including dark *J*–*V*, light-intensity-dependent *V*_oc_, transient photocurrent (TPC), transient photovoltage (TPV), electrochemical impedance spectroscopy (EIS), and Mott–Schottky (M–S) measurements. Figure 5a gives the dark *J*–*V* curves of investigated PSCs. In general, the dark *J*–*V* curve of a solar cell is dominated by the diode law. Based on it, the *J*_0_ can be estimated from the intercept at zero voltage of the linear regression from semi-logarithmic *J*–*V* plot in the region close to the diode turn-on voltage [19, 23, 57]. In general, *J*_0_ is governed by recombination of charge carriers in the cell, wherein a low *J*_0_ is always companied by the suppressed recombination. Herein, we can see that the PSC based on CsPbIBr_2_ film with interfacial voids exhibits much smaller *J*_0_ (1.14 × 10^−7^ mA cm^−2^) than the one without interfacial voids (1.37 × 10^−5^ mA cm^−2^), indicating the weaker recombination of charge carriers in it. It should be noted that although the larger grains and higher crystallinity can partly decrease *J*_0_ of the PSC based on CsPbIBr_2_ film with interfacial voids, we believe that the interfacial voids may dominantly contribute to its much smaller *J*_0_. This is because the PSC exhibits the similarly lower dark current under high bias voltages such as 1.5 V.

Figure 5b presents the plots of *V*_oc_ as a function of incident light intensity for the concerned PSCs. One can see that the *V*_oc_ improves with the logarithmic light intensity monotonically, revealing the dominant trap-assisted Shockley–Read–Hall recombination in both PSCs [9, 23, 30, 31]. In addition, the PSC based on CsPbIBr_2_ film without interfacial void shows a slope of 2.08 *k*_B_*T*/*q*, while the one with voids yields a smaller slope of 1.70 *k*_B_*T/q* that is more close to 1 *k*_B_*T*/*q*, where *q* is the electric charge, *T* is temperature, and *k*_B_ is the Boltzmann constant. These results further confirm that the recombination of charge carries has been greatly suppressed in the PSC based on CsPbIBr_2_ film with interfacial voids.

Figure 5c gives the TPC curves of the corresponding PSCs, which can reflect the extraction and transport properties of charge carriers. The photocurrent decay constants are estimated to be 2.64 and 1.83 μs for the PSCs based on CsPbIBr_2_ film without and with interfacial voids, respectively. These results mean that the interfacial voids do not damage the extraction and transport of charge carriers [18, 19, 51], even though they induce significantly decreased dark current density. Instead, the extraction and transport of charge carriers are improved slightly, which probably benefit from the larger grains and higher crystallinity of CsPbIBr_2_ film with interfacial voids that endow its better transport ability for photon-generated carriers. At the same time, the recombination of charge carriers in the PSCs is monitored by TPV measurements [18, 19, 51]. The results in Fig. 5d indicate that the PSC based on CsPbIBr_2_ film with interfacial voids has the slower photovoltage decay (1.06 ms) than the one without interfacial void (0.79 ms), which supports the suppressed recombination of charge carriers in the former again.

Furthermore, EIS tests were performed on the fabricated PSCs to estimate recombination resistance (*R*_rec_) of charge carriers in them [14, 17, 25]. The Nyquist plots that were recorded at a forward bias voltage of 1.0 V under dark condition are given in Fig. 5e. The clear *arc* is involved to carrier recombination, reflecting the recombination resistance *R*_rec_. The larger *R*_rec_ of the PSC based on CsPbIBr_2_ film with interfacial voids is well accordance with the suppressed recombination of charge carriers in it. On the other hand, the M–S results in Fig. 5f indicate that the built-in potential (*V*_bi_) of the PSC based on CsPbIBr_2_ film with interfacial voids is 1. 56 V, which is much higher than that of the one without interfacial void (*V*_bi_ = 1.30 V). A larger *V*_bi_ not only provide higher driving force to dissociate photo-generated carriers, but also contribute to form a wider depletion region for suppressing recombination of charge carriers in the PSC, as verified by the dark *J*–*V*, TPV, and EIS results above [15, 18, 30, 31]. As a whole, the higher *V*_bi_ and weaker recombination of charge carriers directly contributes to the record-high *V*_oc_ as well as superior FF of the PSC based on CsPbIBr_2_ film with interfacial voids. And, the higher absorption of CsPbIBr_2_ film as a result of intense light scattering effect of interfacial voids gives rise to its higher *J*_sc_. Therefore, the PCE of the PSC based on CsPbIBr_2_ film with interfacial voids is improved significantly.

## Conclusions

We demonstrate a new interfacial engineering for carbon-based, inorganic CsPbIBr_2_ PSC by manufacturing interfacial voids between TiO_2_ ETL and CsPbIBr_2_ film. The interfacial voids contribute to better absorption, higher *V*_bi_, and smaller *J*_0_ of the PSC, as results of enhanced light scattering in CsPbIBr_2_ film, boosted crystal quality of CsPbIBr_2_ grains, and significant suppressed recombination of charge carriers. Consequently, the optimized PSC achieves the PCE of as high as 10.20% coupled with the *V*_oc_ of 1.338 V. Such *V*_oc_ represents the best value among CsPbIBr_2_ PSCs reported earlier. More promisingly, the non-encapsulated PSC can remain ~ 97% and ~ 94% of its initial PCE after being heated at 85 °C for 12 h or storing in ambient atmosphere with relative humidity of 30–40% for 60 days, respectively, suggesting its excellent stability against light, thermal, and humidity stresses.

## Electronic supplementary material

Below is the link to the electronic supplementary material.

Supplementary material 1 (PDF 571 kb)
