# Supplementary material for: Interfacial Voids Trigger Carbon-Based, All-Inorganic CsPbIBr2 Perovskite Solar Cells with Photovoltage Exceeding 1.33 V
Source: Nanomicro Lett. 2020 Apr 6;12:87. doi: 10.1007/s40820-020-00425-1 (PMC7770772; doi:10.1007/s40820-020-00425-1)
Supplement: Supplementary file 1 — Supplementary material 1 (PDF 571 kb) [file 40820_2020_425_MOESM1_ESM.pdf]

Supporting Information for

Interfacial Voids Trigger Carbon-Based, All-Inorganic $CsPbIBr_2$ Perovskite Solar Cells with Photovoltage Exceeding 1.33 V

Weidong Zhu[1,*], Zeyang Zhang[1], Dandan Chen[2], Wenming Chai[1], Dazheng Chen[1], Jincheng Zhang[1], Chunfu Zhang[1,*], Yue Hao[1]

[1]State Key Discipline Laboratory of Wide Band Gap Semiconductor Technology & Shaanxi Joint Key Laboratory of Graphene, School of Microelectronics, Xidian University, Xi'an 710071, People's Republic of China

[2]College of Science, Xi'an Shiyou University, Xi'an, Shaanxi 710065, People's Republic of China

Weidong Zhu and Zeyang Zhang contributed equally to this work

*Corresponding authors. E-mail: wdzhu@xidian.edu.cn (Weidong Zhu); cfzhang@xidian.edu.cn (Chunfu Zhang)

Supplementary Figures and Table

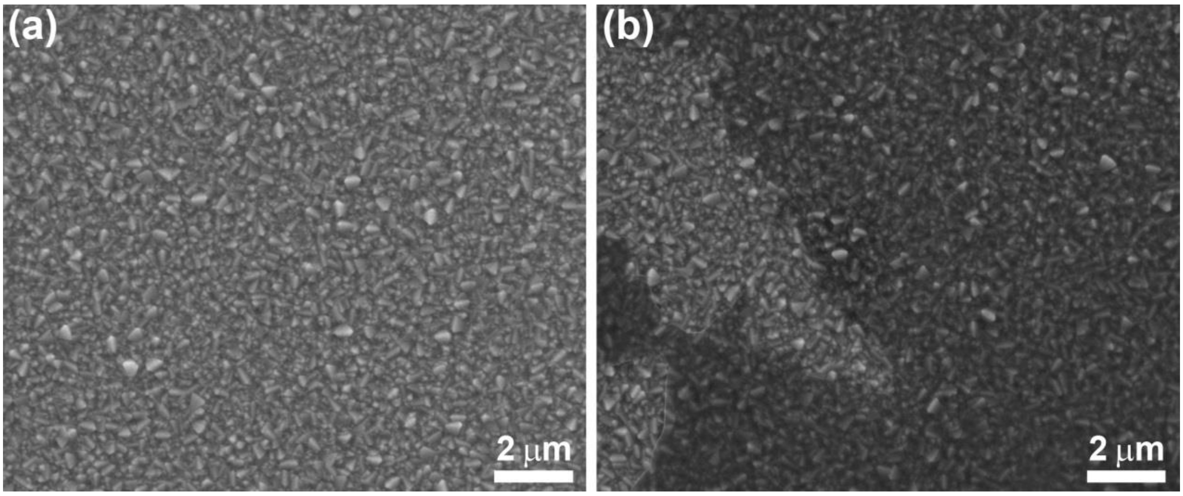

Fig. S1 SEM images of FTO/TiO_2 substrates: **a** without and **b** with loading of PEAI species. The dark area in panel **b** indicates the loading of PEAI species

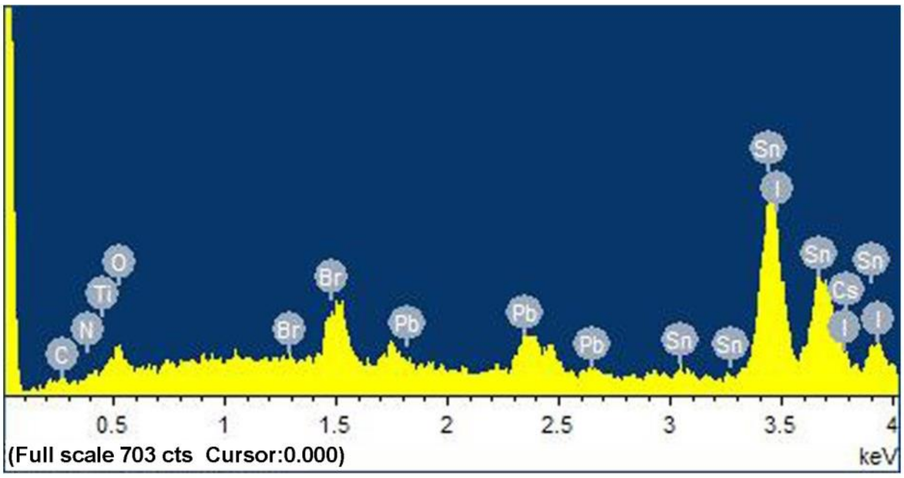

Fig. S2 EDS spectra of $CsPbIBr_2$ film deposited on FTO/TiO_2 substrate with loading of PEAI species.

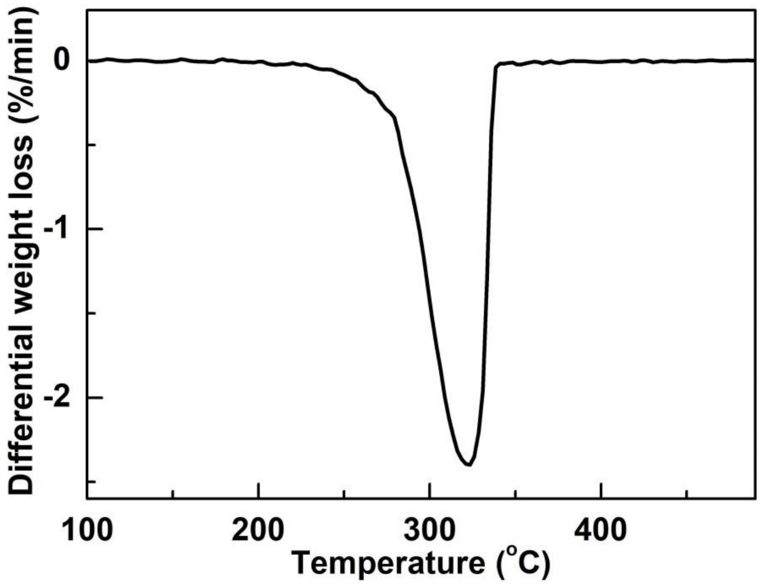

Fig. S3 Differential thermogravimetric curve PEAI powder as a function of applied temperature

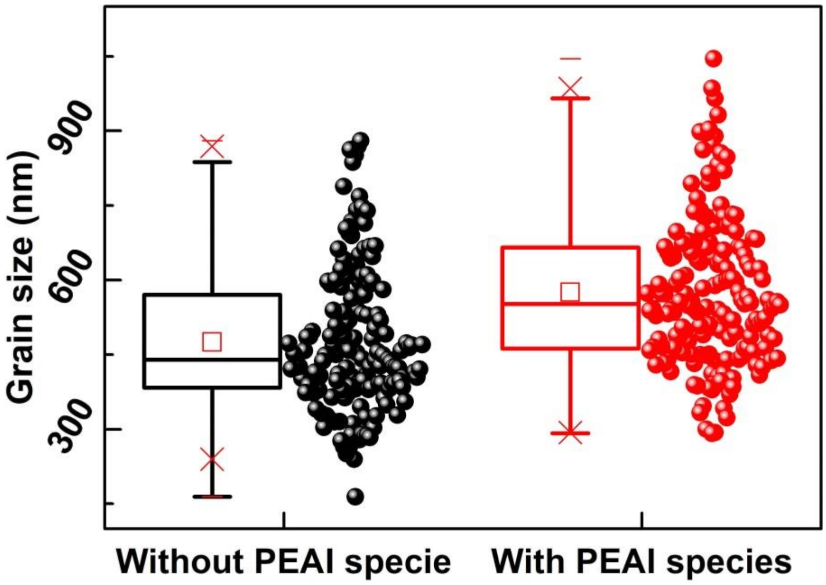

Fig. S4 Statistical grain sizes of $CsPbIBr_2$ films deposited on FTO/TiO_2 substrates without and with loading of PEAI species

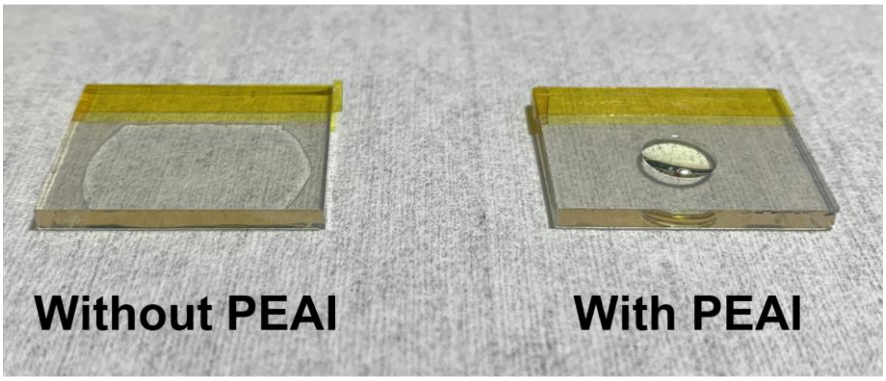

Fig. S5 Optical images of $CsPbIBr_2$ precursor on FTO/TiO_2 substrates without and with loading of PEAI species

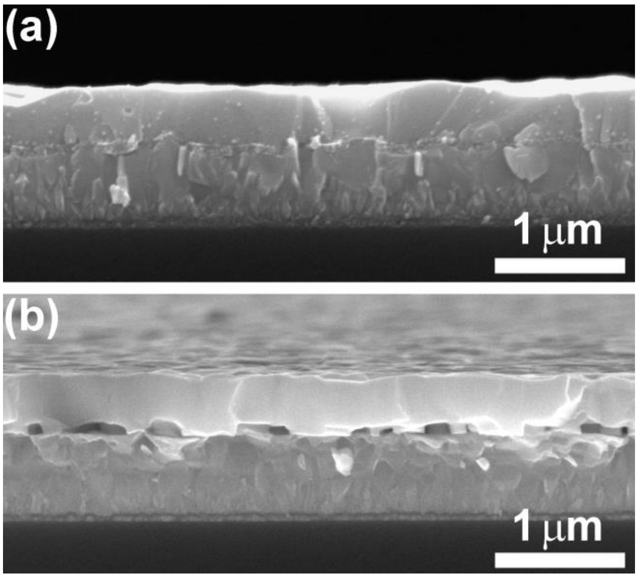

Fig. S6 High-resolution cross-sectional SEM images of $CsPbIBr_2$ films deposited on FTO/TiO_2 substrates: **a** without and **b** with loading of PEAI species

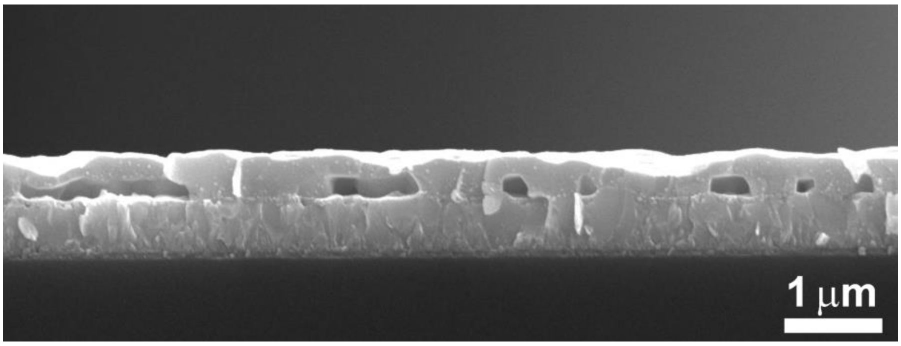

Fig. S7 Cross-sectional SEM image of $CsPbIBr_2$ film prepared with 10 mg mL^{-1} PEAI solution

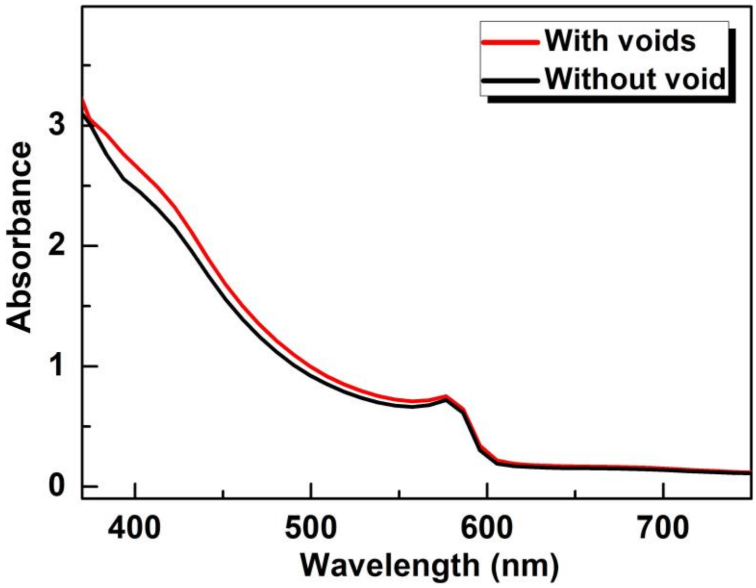

Fig. S8 UV-vis absorption spectra collected from the side of FTO glass for $CsPbIBr_2$ films without and with interfacial voids, respectively, wherein the detection beam streams from the top surface of $CsPbIBr_2$ films

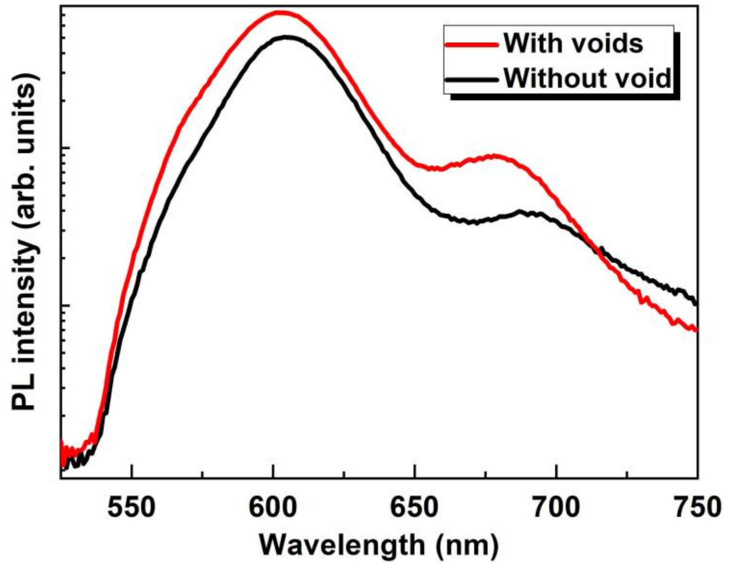

Fig. S9 Logarithmic-scale steady-state PL spectra of $CsPbIBr_2$ films without and with interfacial voids on insulating glass substrates instead of FTO/TiO_2 substrates

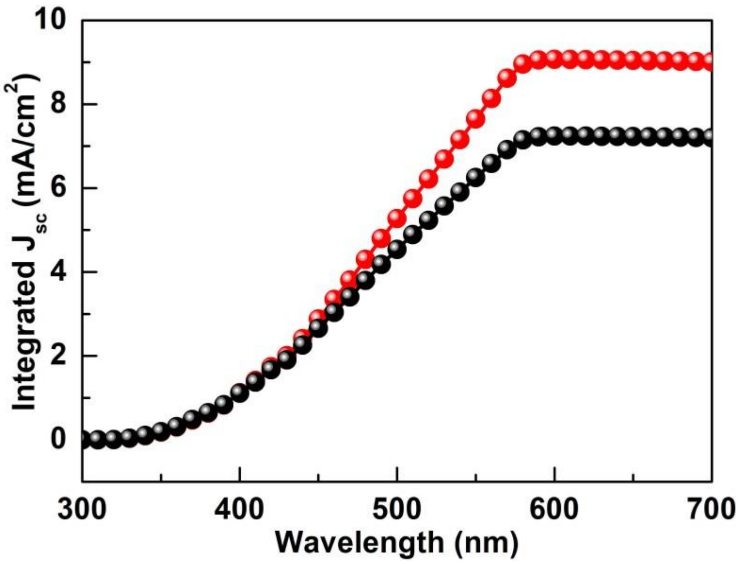

Fig. S10 Integrated J_{sc} curves of the champion cells based on $CsPbIBr_2$ films without and with interfacial voids

Table S1 Fitted parameters of (100) and (200) XRD peaks of $CsPbIBr_2$ films without and with interfacial voids

Samples	Peak of (100) plane			Peak of (200) plane		
	Position	Intensity	FWHM	Position	Intensity	FWHM
Without void	15.13°	1072	0.163°	30.44°	1321	0.190°
With voids	15.14°	1680	0.126°	30.42°	1917	0.177°
